# Reviews

**Published:** 1863-11

**Authors:** 


### REVIEWS.

*A Report on Hospital Gangrene, Erysipelas and Pyæmia, as observed in the Departments of the Ohio and the Cumberland, with cases appended.* By M. GOLDSMITH, *U. S. V. Published by permission of the Surgeon-General U. S. A. Louisville:* BRADLEY & GILBERT, *corner of Third and Green streets,* 1863.

This report is an exceedingly interesting and important one, full of statistical evidence and eminently worthy the consideration of all interested in the subjects it considers. The points of semblance and of difference in these diseases are very plainly shown. Cases are introduced showing with great clearness the origin and causes of these diseases, in many cases, For the purpose of giving our author's own description of the disease, known as Hospital Gangrene, we will quote a paragraph or two embracing his description of the disease, and perhaps also part of his directions for the application of bromine, which seems certainly to have proved a sovereign remedy:

"The cases of hospital gangrene, which have been treated in the hospitals at this place, have presented some constancy in most of their characteristics; and in order that the true nature of the disease may be apparent to the Surgeon-General, I will state the appearances commonly presented:

1st.—The gangrenous affection presented a tolerably constant tendency to the assumption of a somewhat circular form. This tendency, however, was frequently interrupted by the varying effects of the special remedy used. Thus, when the skin was not undermined to any great extent, the disease was commonly arrested immediately, and the ulcer left, presented the usual circular form; but when the skin was much undermined, or undermined to unequal extent, at different points in the circumference, the disease was not arrested as promptly at one point as at another, and thus, eventually, the circular form was lost. Then, again, the gangrenous erosion had sometimes an irregular elongate form, coinciding with the original shape of the wound. Sometimes the gangrene attacked the walls of a wound passing through a limb, and presented itself as the sloughing core of a ball wound, the pulps protruding from the aperture of both entrance and exit.

2d.—The spread of the gangrene seemed pretty generally to be influenced by the succulency of the tissues. Thus, when it commenced on the surface in a superficial wound or ulcer, it generally spread most rapidly in the skin and cellular planes. The disease continuing, the muscular substance would next be attacked. Dense fasciæ, as the fascia lata, and tendons, resisted the influence much longer; on the whole, the bones suffered more than tendons. Another reason why the disease spread laterally rather than deeply, and of consequence more in cellular planes, and less rapidly towards the deeper tissues, is, that the sloughs could be more readily detached from the exposed parts of the gangrenous surface so as to allow the effectual application of the remedy, while it was difficult to detach the sloughs underlying the skin, and, of course, difficult to mix the remedy with the sloughs—the *sine qua non* of its curative agency. These causes have operated so decidedly in modifying the form and appearances of the granulating sores left on the subsidence of the gangrene, that many of the numerous surgeons visiting this place, to see for themselves the effects of the treatment instituted, have found it difficult to realize that they were looking upon the ravages of hospital gangrene, unless it so happened that I could show them some cases in which the disease was in progress.

3d.—The sloughs were variant in their consistence, and this variance ran from tolerably firm escars to diffluent pulps. The consistence of the sloughs coincided rather with the consistence of the tissues sloughing. The sloughs of skin were soft, swollen, tolerably coherent masses. The sloughs of cellular tissue were soft, flocculent, yielding more abundantly a dirty yellowish fluid. The sloughs of muscles were firmer, less pulpy, more coherent. In some of the cases, and especially in those in which the process was slower, skin, cellular substance, and muscles, seemed to melt away into mere diffluent matter, the product of the destruction of each of the several tissues in these cases being nearly alike.

4th.—The sloughs were commonly of a dirty greyish hue, those of the skin being, in most instances, somewhat darker than those of the cellular substance. The variations of color appeared to be influenced more by the quantities of altered blood in the tissues than by any other condition.

5th.—In all of the cases there was present a most pungent and intolerable fetor. In some instances the pungency of the gaseous effluvia was so great as to produce a persistent smarting in the eyes and the nares of the persons engaged in dressing the sores. The odor would often fill the whole ward. This fetor, in greater or less intensity, was the almost constant attendant upon the gangrenous process, appearing when it began, continuing as it continued, and ending when it ended. So constant was this coincidence that those who treated the case came to regard the disappearance of the fetor as the reliable evidence of the arrest of the disease; the presence of it, as the signal of the commencement of the process. This odor was peculiar; it was not the sickish odor which is often perceived in suppurating or ulcerating wounds, nor the odor of common gangrene, or of common putrefaction of dead animal matter, but an odor peculiar, recognizable with the nose, but not admitting of description."

"Later experience with the bromine in my own hands satisfies me that the following directions for its application are sufficient for almost any contingency:

1st. *Preparation.* If the gangrenous sore is large, or the patient intolerant of pain, chloroform or ether should be administered.

2d. The surgeon should be provided with basins, sponges, dry lint, blotting paper if at hand, a stout rubber or a glass urethral syringe, a small measuring glass, some *pure bromine,* a pair of foreceps, probe scissors, and a spatula or the handle of a scalpel.

3d. The patient being prepared, the surgeon should, with forceps and scissors, remove all the sloughs so far that some bleeding points are exposed, The bleeding having ceased, or been arrested by the touch of the bromine he next scrapes away the fluid putrilage or purulent fluid bathing the surface of the sore; he now turns up the edges of the skin, and, with the handle of the scalpel, removes all the pultaceous matter underlying the skin. The flocculent pulps adherent to the surfaces underneath are now removed with the scissors and forceps. The same proceedings are practised in the cellular planes between muscles. The surfaces are now to be dried, first with lint or tow, and finally with blotting paper, or any dried paper pulp. The bromine is now poured into the glass measure partly filled with water. The syringe, or a pipette, is next thrust through the water into the layer of bromine, and this is drawn up into the syringe or pipette; with this instrument the bromine is applied, first to the cavities between the muscles, next under the skin, next to the exposed surfaces under the sore.

This application of the bromine coagulates and hardens the soft flocculent pulp, and gives the fluid parts of the putrilage the consistence of brain substance. The scissors and scalpel are again put into requisition; the gangrenous portions may now be easily removed, and when it is done, from under the skin, from the intermuscular spaces, and from the exposed surfaces of the sore, the bromine should be re-applied. Where the gangrene attacks the elongated track of a ball-wound—traversing a limb, for example—a piece of candle-wick, threaded in an eyed probe, should be saturated with the bromine and passed through the wound."

We regret not being able to publish more of the report, which is certainly a most valuable contribution to the knowledge of the profession, upon this subject. The volume is composed of nearly a hundred pages, and is devoted to a full and complete consideration of the topics introduced. Much careful observation has been made by the author, and much truth has apparently been discovered and elucidated.

For sale in this city by the dealers in medical books.

---

*Synopsis of the Course of Lectures on Materia Medica and Pharmacy. Delivered in the University of Pennsylvania, with three Lectures on the Modus Operandi of Medicines, by* JOSEPH CARSON, M. D., *Philadelphia:* BLANCHARD & LEE, 1863.

In this book, we have what may be termed the headings of the various subjects which are to be carefully studied, and in some instances brief descriptions and statements of facts. Students who attend lectures at the University of Pennsylvania, will find the book an indispensable outline to the course of lectures upon Materia Medica and Pharmacy; while students everywhere will find it a guide to the leading facts and principles comprised in this extensive subject. This synopsis, as here given, has sometimes been bound with blank leaves, and in any case, blank leaves can be supplied to the frame work here published, which could be filled in with notes taken at the time lectures are delivered, or by reference to works upon the subject. This frame work requires to be filled up in some such way before it appears at all complete.

The three lectures upon the Modus Operandi of Medicines, contain new suggestions, and comprise the results of investigation with respect to the part taken by the nervous system in the action of medicines, and the proofs of absorption. The names of the medicinal articles and their preparations, have been made to conform to the United States Pharmacopœia of 1863. This book is eminently worthy the attention of medical students, and may be obtained of the book merchants in this city.

